# Seeing the world through the eyes of cultured cells

**DOI:** 10.1016/j.cmet.2025.01.027

**Published:** 2025-03-12

**Authors:** Joycelyn Tan, Guy B. Kunzmann, Sam Virtue, Jason R. Cantor, Daniel J. Fazakerley

**Affiliations:** 1 Metabolic Research Laboratories, Institute of Metabolic Science, University of Cambridge, Cambridge CB2 0QQ, UK; 2 Morgridge Institute for Research, Madison, WI, USA; 3 Department of Biochemistry, University of Wisconsin-Madison, Madison, WI, USA; 4 No current affiliation; 5 Department of Biomedical Engineering, University of Wisconsin-Madison, Madison, WI, USA; 6 University of Wisconsin Carbone Cancer Center, Madison, WI, USA

## Abstract

The metabolic environment experienced by cultured cells often differs from physiological conditions. Here we highlight the effects that the microenvironment can have on cultured cell behavior, and advocate for researchers to re-evaluate culture practices to enhance the relevance and translational potential of *in vitro* studies.

Cell culture provides an accessible and flexible system to investigate fundamental cell biology and drug sensitivity. Studies of mammalian cells *in vitro* operate under the implicit assumption that they generate results reflective of cellular behaviour *in vivo*. However most *in vitro* studies rely on culture protocols that create metabolic environments far removed from conditions that cells may face in the body. Further consideration into how such environments affect cell behaviour could aid the translation of findings from *in vitro* studies. Here, we focus on the contributions of medium composition and oxygen provision, while noting that other aspects of the *in vitro* environment, from cell attachment substrate (mechanical stresses) to various co-culture methods (intercellular interactions), also impact cellular metabolism.

## What is a healthy diet for cells?

In the early 1950s, Harry Eagle defined Basal Medium Eagle (BME), which contained a minimum set of essential nutrients at concentrations he deemed as optimal for the survival and proliferation of HeLa cells^1^. Notably, most basal synthetic media recipes can be traced back to his pioneering work, including RPMI 1640 (RPMI) and Dulbecco’s Modified Eagle Medium (DMEM). However, these recipes were not created to simulate the composition of any physiologic biofluid. DMEM contains 25 mM glucose and 15 proteinogenic amino acids (10.7 mM total), while RPMI contains 11.1 mM glucose and 19 amino acids (6.6 mM total) – both formulations lack defined lipids and contain several vitamins at concentrations that in large part are much higher than those found in plasma. Collectively, these two synthetic media, which remain the workhorses across most *in vitro* studies, provide cells with nutrient “diets” that have somewhat limited relevance to human health and disease.

## Macronutrients

Fats: One striking difference between human plasma and existing *in vitro* models is the absence of defined lipids in most synthetic basal media. In most cases, serum supplementation serves as the only supply of lipophilic species, which in turn, are typically unaccounted for and may further vary both by source (e.g., fetal bovine versus calf) and lot. Ultimately, cells cultured in media containing 5 to 20% serum receive substantially less total lipid than cells *in vivo*. Therefore, cultured cells can display highly distinct lipid profiles versus those in animals^2^, including marked differences among polyunsaturated fatty acids (PUFA) and monounsaturated fatty acids. Furthermore, prior work reported that murine fibroblasts and hepatoma cells grown in culture accumulated Mead acid (20:3n-9), a *de novo* synthesized PUFA that can act as a clinical indicator of essential fatty acid deficiency in humans^3^. In addition, others found that limited exogenous availability of the essential fatty acid arachidonic acid (20:4ω-6) altered the eicosanoid profile of RAW 264.7 macrophages and enhanced their inflammatory activity^4^. Despite the potential benefit of improving *in vitro* modelling capacity, incorporation of defined lipids into culture media remains a challenge for several reasons: (I) comprehensive and quantitative characterization of lipidomes for biofluids are needed; (II) traditional methods for removing lipids from serum supplements can also deplete critical growth factors and hormones; (III) low intrinsic solubilities make it difficult to precisely add lipophilic species to aqueous media; and (IV) most physiologic lipids are not readily available to purchase.

Carbohydrates: Glucose is often the primary carbohydrate substrate provided by synthetic culture media. Nonetheless, defined glucose concentrations in DMEM (25 mM) and RPMI (11.1 mM) are far higher than normal fasting blood glucose levels (3.9 to 5.6 mM). Upon cell entry, glucose is typically catabolized over a set of biochemical pathways that comprise central carbon metabolism, resulting in the production of energy and various metabolic precursors. Of note, recent evidence has shown that culture in media containing “high” versus more physiologic glucose concentrations can impact cell metabolism, including effects on respiration rates and mitochondrial dynamics^5,6^.

Protein: For most cases, the amino acid concentrations defined in traditional synthetic media also markedly differ relative to those in biofluids such as plasma. For example, RPMI and DMEM each lack defined alanine, the second most abundant amino acid (after glutamine) found in human blood. By contrast, the defined glutamine concentrations for DMEM (4 mM) and RPMI (2 mM) are each several-fold higher than those found in normal plasma (0.4 to 0.7 mM). Glutamine is typically incorporated into media in either its free form or as part of an alanyl-glutamine dipeptide supplement (GlutaMAX). Although GlutaMAX may offer increased glutamine stability, using this supplement also introduces an equivalent but often unaccounted for quantity of alanine upon intracellular cleavage of the dipeptide. Notably, recent work has revealed that the differential availability of amino acids such as alanine can impact the behaviour of cultured cells^7^. For example, CRISPR screening in RPMI versus Human Plasma-Like Medium (HPLM) revealed that glutamic-pyruvic transaminase 2 (*GPT2)* and both components of the mitochondrial pyruvate carrier *(MPC1* and *MPC2)* were differentially required to support cell growth in RPMI – conditional CRISPR phenotypes each traced to the unique inclusion of defined alanine in HPLM^8^.

## Other metabolites and micronutrients

Efforts to assess the possible impacts of medium composition on cell behavior have shown that the availability of vitamins, minerals, and other metabolites may also influence cell metabolism and drug sensitivity^7,8^. Prior work in donor T cells revealed that culture in HPLM versus RPMI markedly changed global gene expression and activation upon stimulation, an effect traced to a difference in exogenous Ca^2+^, which is sub-physiologic in RPMI^9^. Moreover, culturing blood cancer cells in HPLM versus RPMI revealed an unforeseen example of metabolic regulation linked to uric acid – a ‘waste product’ uniquely defined in HPLM that is also present at 10-fold higher levels in human versus mouse blood^7^.

## Oxygen

The role of oxygen tension in cell culture has received considerably less recent attention relative to medium composition, perhaps since pericellular oxygen levels can be more difficult to measure. Conventional *in vitro* models are often considered hyperoxic given the lack of oxygen control in standard incubators, which expose cells to ∼18.6% oxygen (140 mmHg). By contrast, the oxygen levels in blood circulation (50-70 mmHg) and across various normal human tissues (5-100 mmHg) are typically far lower. However, rates of oxygen diffusion in air are considerably faster than through media. For cell types that commonly grow as two-dimensional monolayers in traditional culture systems, these differential diffusion kinetics may lead to oxygen concentration gradients across the depth of the media, whereby oxygen levels are highest at the surface and lowest near monolayers consuming oxygen. If the cellular rate of oxygen use exceeds that of oxygen diffusion across the media layer, pericellular hypoxia can ensue^10^. Therefore, in some cases, adherent cells may effectively be exposed to oxygen-limiting conditions in standard incubators, and in turn, exhibit phenotypes consistent with cellular responses to hypoxia^10^. By reducing the culture volume to decrease the oxygen diffusion distance, such responses can be mitigated, leading to positive effects on phenotypes such as induced pluripotent stem cell-derived hepatocyte differentiation^10^.

Multiple factors likely contribute to pericellular oxygen tensions, including cellular rates of oxygen uptake, cell density/confluence, and the depth of the overlying culture medium. In addition, rates of oxygen diffusivity are shaped by several parameters such as temperature, ionic strength of the culture medium, and atmospheric pressure. Together, putative oxygen concentrations within the “bulk” incubator can be a poor indicator of the oxygen tension experienced by cultured cells, as intended oxygen levels found at the cell monolayer may not be achieved by simply modifying the set point for incubator gas composition. However, selecting relevant oxygen tensions *in vitro* will require improved understanding of the respective *in vivo* environment, including the possibility of tissue- and organ-dependent oxygen gradients.

## Growth conditions in standard batch culture are not fixed

Accumulating evidence demonstrates that medium composition and oxygen provision can each affect cell behaviour. Most *in vitro* studies are carried out in a batch format (i.e., dishes, plates, or flasks). Cells cultured in these formats are typically passaged (proliferating cells) or fed (non-proliferating cells) every 48 to 96 hours. Sharp changes to the metabolic environment are inherent to such protocols, as the resulting medium exchange “resets” nutrient availability and dissipates established oxygen gradients. Moreover, while we can at least in part control culture conditions at inoculation, medium composition and pH change over time, as cells gradually consume or secrete metabolites between passages or re-feeding. This real-time environmental variation may lead to unanticipated and unaccounted cellular responses or adaptations, including effects on nutrient preferences and metabolic pathway activities. For example, GLUT1 has a Michaelis constant (Km) for glucose transport of ∼1-2 mM, and in turn, likely operates at near maximum velocity at or above normal fasting blood glucose concentrations. However, if extracellular glucose levels approach or fall below this Km value, the ensuing decrease in relative glucose uptake may force increased cellular reliance on alternative carbon sources.

The dynamics of metabolite availability in traditional batch culture formats are likely impacted by several factors, from initial medium composition and culture volume to the cell type and seeding density. Of note, when Eagle first reported Minimum Essential Medium (MEM), his choice to broadly boost amino acid levels versus those he had defined for BME – just a few years earlier – was in part motivated by the potential to maintain cell cultures over longer periods without re-feeding^11^. This anecdote not only highlights historical appreciation for the uncontrolled changes in medium composition inherent to batch culture, but also the distinction in factors that contributed to classic synthetic media design relative to the modelling objectives of more recently described physiologic media^12^.

## So, should I repeat all my cell culture experiments?

**Figure 1:**
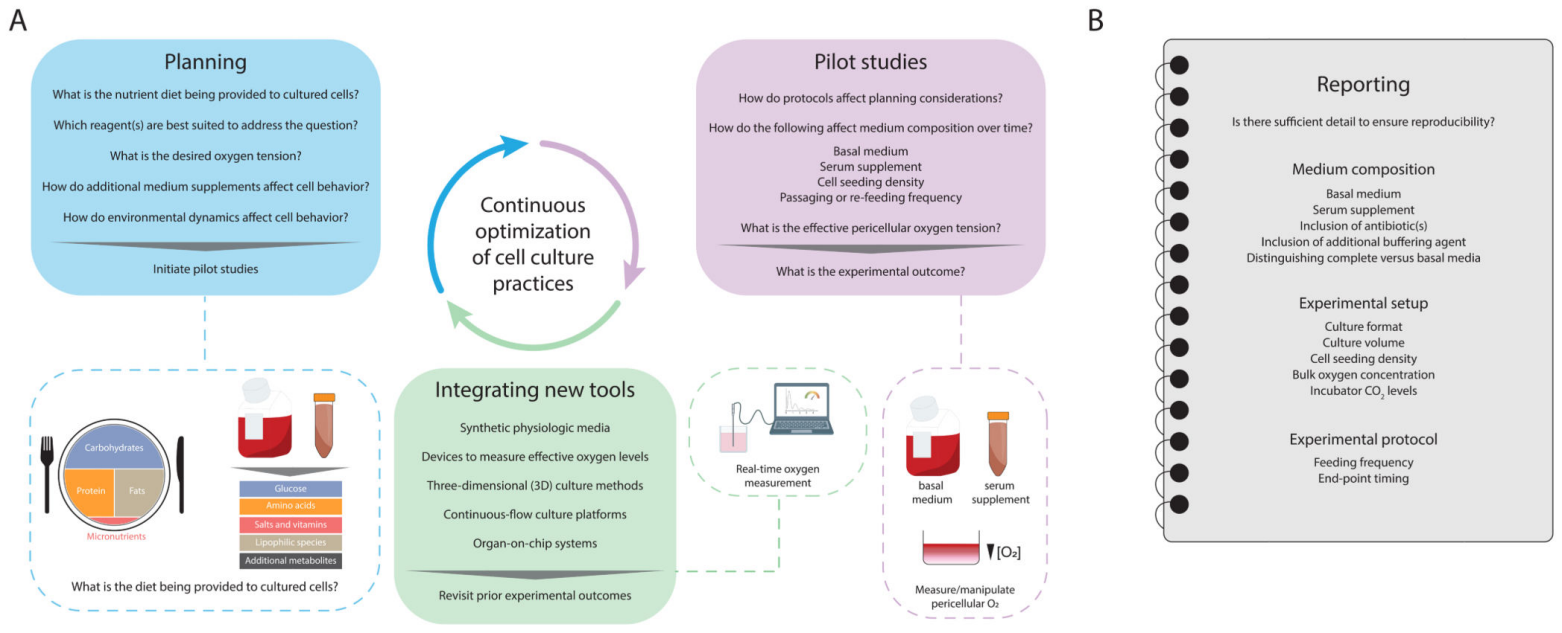
***A***, Proposed workflow for continuous optimization of cell culture models. ***B***, Comprehensive reporting of various cell culture parameters can help to improve experimental reproducibility.

No, not necessarily. Studies performed using traditional *in vitro* models have led to many important discoveries in fundamental cell biology and advances in drug development. Importantly, it is not realistic to expect to fully capture the intricacy of conditions faced by cells *in vivo*. However, scientists should not rest on historical precedence given the growing appreciation that metabolic conditions can affect experimental outcomes. For instance, it may be worth asking why studies in specific cell types often remain tied to a particular set of culture parameters such as basal medium and serum supplement. How were these choices first established? Why have they remained largely static despite recent technological advances and accumulating evidence that environmental factors contribute to cell biology? Are they optimal based on the specific question(s) being addressed? Moving away from an autopilot-like approach to cell culture standards holds immense potential (Figure 1A), with scientists having far greater control over the design of *in vitro* models aided by recent efforts in tool development. These include the integration of devices that enable more precise measurements of pericellular oxygen levels within incubators and the systematic development of synthetic “physiologic media” designed to more closely mimic nutrient compositions found in biofluids such as blood (human^7,13^ or mouse^14^) and bulk interstitial fluid isolated from murine pancreatic tumors^15^. For example, beyond several marked differences in concentration among components common to most synthetic media, HPLM also provides more than 30 components not otherwise defined in either RPMI or DMEM, including alanine and a variety of metabolites that could serve as alternative carbon sources such as lactate and acetate.

Such efforts to advance modelling capacity do not inevitably refute insights from studies of cells cultured using more traditional conditions and formats. Instead, understanding cell-environment interactions might, in some cases, clarify why prior studies failed to translate in animal models or patients – offering opportunities to identify new insights previously masked in traditional models. In addition, while it is certainly valid to ask whether ongoing efforts intended to advance *in vitro* modelling capacity facilitate phenotypes more reflective of those in humans, it should be recognized that putative options to directly address this question are impractical if not unattainable in the context of most studies. Nonetheless, we reason that models more closely simulating metabolic conditions found in the body are likely to yield data more reflective of *in vivo* cell biology.

Given the growing range of possible tools and methods that may be leveraged in designing *in vitro* models, a more comprehensive reporting of culture protocols will be needed to facilitate experimental reproducibility. Beyond typical descriptions of synthetic media, serum percentages, and incubator temperatures, we suggest that authors also consistently report culture formats (e.g., dish diameters or flask sizes), culture volumes, seeding densities, oxygen tensions, use of antibiotics or buffers in media, incubator CO_2_ levels, feeding frequencies, and designations used to distinguish “complete” serum-supplemented from basal media (e.g., RPMI10 or RPMI^+S^) (Figure 1B). The STAR Methods (structured, transparent, accessible reporting) format introduced by Cell Press serves as a useful means for comprehensively reporting reagents, resources, and experimental method details.

With an ever-increasing emphasis on putative translational implications, we contend that the time is right to revisit historical choices regarding the design of *in vitro* models and to also more closely consider the possible impacts of metabolic conditions on cell behavior. Cell culture remains an essential tool across virtually all areas of biology. Thus, we believe that the continued evolution of *in vitro* models, driven by current scientific objectives, rather than the historical motivation of supporting the survival and growth of cells, are necessary for advancing the utility of cell culture.
